# Purinergic Receptors in the Airways: Potential Therapeutic Targets for Asthma?

**DOI:** 10.3389/falgy.2021.677677

**Published:** 2021-05-31

**Authors:** Rebecca J. Thompson, Ian Sayers, Katja Kuokkanen, Ian P. Hall

**Affiliations:** ^1^Division of Respiratory Medicine, Nottingham Biomedical Research Centre, National Institute for Health Research, University of Nottingham Biodiscovery Institute, University of Nottingham, Nottingham, United Kingdom; ^2^Orion Corporation, Orion Pharma, Research and Development, Turku, Finland

**Keywords:** purinergic signaling, purinergic receptor, lung, airway, bioinformatics, gene expression, asthma

## Abstract

Extracellular ATP functions as a signaling messenger through its actions on purinergic receptors, and is known to be involved in numerous physiological and pathophysiological processes throughout the body, including in the lungs and airways. Consequently, purinergic receptors are considered to be promising therapeutic targets for many respiratory diseases, including asthma. This review explores how online bioinformatics resources combined with recently generated datasets can be utilized to investigate purinergic receptor gene expression in tissues and cell types of interest in respiratory disease to identify potential therapeutic targets, which can then be investigated further. These approaches show that different purinergic receptors are expressed at different levels in lung tissue, and that purinergic receptors tend to be expressed at higher levels in immune cells and at more moderate levels in airway structural cells. Notably, P2RX1, P2RX4, P2RX7, P2RY1, P2RY11, and P2RY14 were revealed as the most highly expressed purinergic receptors in lung tissue, therefore suggesting that these receptors have good potential as therapeutic targets for asthma and other respiratory diseases.

## Introduction

The critical role of intracellular adenosine 5'-triphosphate (ATP) in energy transfer within the cell is well-recognized. Since the first proposal of ATP as a non-adrenergic, non-cholinergic neurotransmitter in 1972 by Geoffrey Burnstock, the hypothesis that extracellular ATP functions as a signaling messenger, termed “purinergic signaling,” has become increasingly accepted. Initially, ATP was thought to only be released as a “danger signal” by damaged and dying cells. However, ATP is now known to be released by healthy cells of virtually all tissue types in a wide array of physiological processes. These range from short-term signaling actions, such as neurotransmission, secretion and acute inflammation, to long-term (trophic) actions, including cellular proliferation, differentiation, motility, and death during development and regeneration. However, purinergic signaling has also been implicated in many pathophysiological conditions, including cancer, neuropathic and inflammatory pain, and neurodegenerative and neuropsychiatric diseases (1).

Given the extensive involvement of purinergic signaling in health and disease, this review will focus specifically on its roles in lung and airway cell biology, and in particular the potential relevance to asthma. Purinergic signaling is known to be important for mucociliary clearance, via stimulating surfactant release, mucin secretion and ciliary beat frequency, and for modulating airway diameter through its actions on airway smooth muscle and the tracheal ring. Purinergic signaling is also involved in the recruitment and activation of immune cells, such as alveolar macrophages, lung dendritic cells and lung mast cells (2). Furthermore, numerous respiratory diseases have been shown to have aberrant purinergic signaling, including asthma, chronic obstructive pulmonary disease (COPD), lung injury and infections, cystic fibrosis, lung cancer, and chronic cough (2). Consequently, purinergic receptors are considered to be promising therapeutic targets for such diseases (3).

ATP is involved directly in airway inflammation (a key hallmark of asthma) through activation of a variety of immune cell types, including eosinophils (4, 5), mast cells (6, 7), dendritic cells (8–10), and alveolar macrophages (11–13). Furthermore, both ATP and adenosine (a breakdown product of ATP) have been shown to have pro-asthmatic roles within the epithelia by stimulating mucin production (14, 15), and adenosine also upregulates fibronectin expression and therefore may be involved in airway remodeling (16). Additionally, a purinergic receptor (ADORA1) has been previously implicated in asthma susceptibility by genome wide association studies (GWAS) and by functional studies (17), thereby further supporting the potential for purinergic receptors as therapeutic targets for asthma. On the basis that receptors with higher expression levels may be more effective targets, this review utilizes bioinformatics approaches to determine the expression profile of purinergic receptors in lung and airway tissue, and explores current evidence implicating specific receptors as potential therapeutic targets for respiratory diseases, with particular reference to asthma.

### Extracellular ATP Acts as a Signaling Messenger by Activating Purinergic Receptors at the Cell Membrane, of Which There Are Three Distinct Receptor Subfamilies

**Figure 1:**
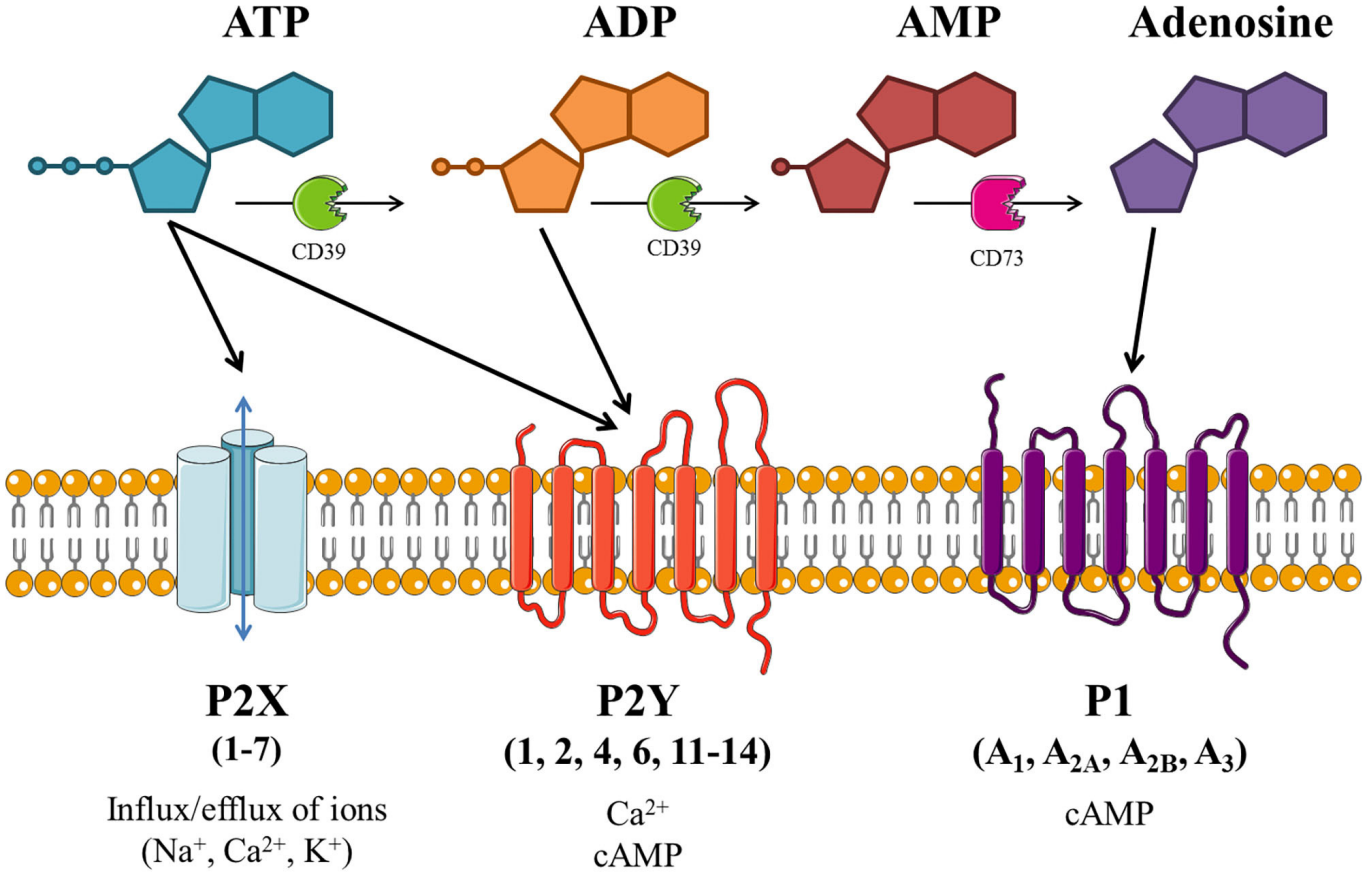
Schematic of purinergic signaling. Extracellular adenosine 5'-triphosphate (ATP) activates P2X and/or P2Y receptors, thus altering intracellular concentrations of ions and/or cyclic adenosine monophosphate (cAMP). ATP can be hydrolyzed by ectonucleotidases (including CD39 and CD73), producing adenosine diphosphate (ADP), adenosine monophosphate (AMP) and adenosine. ADP is also able to activate P2Y receptors, while adenosine activates P1 receptors which modulate adenylate cyclase (AC) activity to alter intracellular cAMP levels. Image is based on Giuliani et al. (18), created with the aid of Servier Medical Art.

As depicted in Figure 1, extracellular ATP can be hydrolyzed by ectonucleotidases on the cell surface into adenosine di- and monophosphate (ADP and AMP, respectively) and adenosine. These breakdown products, alongside the pyrimidine nucleotides uridine tri- and diphosphate (UTP and UDP, respectively), are also involved in purinergic signaling. The effects of purinergic signaling are mediated via “purinergic receptors,” which are expressed on the majority of cell types. There are three distinct subfamilies of purinergic receptor: P1, P2X, and P2Y; with cell types commonly expressing multiple receptor subtypes (18).

P1 receptors are specifically activated by adenosine, and so these receptors are often referred to as adenosine receptors (ADOR). These G protein-coupled receptors (GPCRs) modulate adenylate cyclase (AC) to alter cyclic AMP (cAMP) levels within the cell. There are four subtypes of P1 receptor: ADORA1 and ADORA3 act via G_i/o_ to inhibit AC and lower cAMP levels; whereas ADORA2A and ADORA2B act via G_s_ to activate AC and increase cAMP levels. ADORA2B also acts via G_q_ to activate phospholipase C and subsequently increase intracellular calcium levels (19). ADORA1 and ADORA2A have high potency for adenosine (half-maximal effective concentration (EC_50_) = 1–10 nM and 30 nM, respectively), while ADORA2B and ADORA3 have lower potency (EC_50_ = 1 μM and 100 nM, respectively), as shown in Table 1 (20).

**Table 1:** Purinergic receptor agonists and potency data.

**Purinergic receptor**	**Adenosine**	**ATP**	**ADP**	**UTP**	**UDP**	**UDP-glucose**
ADORA1	1–10 nM					
ADORA2A	30 nM					
ADORA2B	1 μM					
ADORA3	100 nM					
P2RX1		0.1–0.7 μM				
P2RX2		2–8 μM				
P2RX3		~1 μM				
P2RX4		1–10 μM				
P2RX5		0.5 μM				
P2RX6						
P2RX7		2–4 mM				
P2RY1			10 μM			
P2RY2		0.1 μM		0.01 μM		
P2RY4				1 μM		
P2RY6					0.3 μM	
P2RY11		10 μM				
P2RY12			0.1 μM			
P2RY13			0.01 μM			
P2RY14					0.1 μM	0.3 μM

*The half-maximal effective concentration (EC_50_) is given for each extracellular nucleotide that has been shown to activate a given purinergic receptor in humans. Table is based on data from **Jacobson and Muller** (20)*.

P2Y receptors are also GPCRs, of which there are eight subtypes: P2RY1, P2RY2, P2RY4, P2RY6, P2RY11, P2RY12, P2RY13, and P2RY14. The missing numbers pertain to non-mammalian orthologs or receptors deemed unresponsive to nucleotides. P2Y receptors can be activated by a variety of nucleotides with different potencies (EC_50_), as summarized in Table 1, including ATP [P2RY2 (0.1 μM); P2RY11 (10 μM)], ADP [P2RY1 (10 μM); P2RY12 (0.1 μM); P2RY13 (0.01 μM)], UTP [P2RY2 (0.01 μM); P2RY4 (1 μM)], UDP [P2RY6 (0.3 μM); P2RY14 (0.1 μM)], and UDP-glucose [P2RY14 (0.3 μM)] (20). Most P2Y receptors (P2RY1, P2RY2, P2RY4, P2RY6, and P2RY11) act via G_q_ to activate phospholipase C, leading to increased intracellular calcium levels and protein kinase C activation, whereas P2RY12, P2RY13, and P2RY14 act via G_i/o_ to inactivate AC, therefore reducing cAMP levels and protein kinase A activation. P2RY11 can also act via G_s_ to activate AC and increase cAMP levels (19).

Conversely, P2X receptors are ATP-gated ion channels permeable to calcium, sodium and potassium ions. Each of the seven subtypes (P2RX1–7) has a topology of intracellular termini and two transmembrane (TM) domains, with TM1 involved in channel gating and TM2 lining the channel pore. The large extracellular loop contains regions of acidic residues thought to attract cations to the channel. The functional ion channel is formed from three subunits, and can be homo- or heterotrimeric, with three binding sites for ATP which must all be occupied for the channel pore to open (21, 22). P2RX1 and P2RX3 have high potency for ATP (EC_50_ = 1 μM) and are rapidly activated and desensitized. P2RX2 and P2RX4 have lower ATP potency (EC_50_ = 10 μM) and desensitize slowly, with sustained depolarizing currents. P2RX7 has very low ATP potency (EC_50_ = 2–4 mM) with little or no desensitization. Conversely, P2RX5 and P2RX6 are thought to be non-functional or require heteromerization to form a functional channel [see Table 1 (20)].

### Online Bioinformatics Provide Purinergic Receptor Expression Data in Lung Tissue and Identify Promising Therapeutic Targets

**Figure 2:**
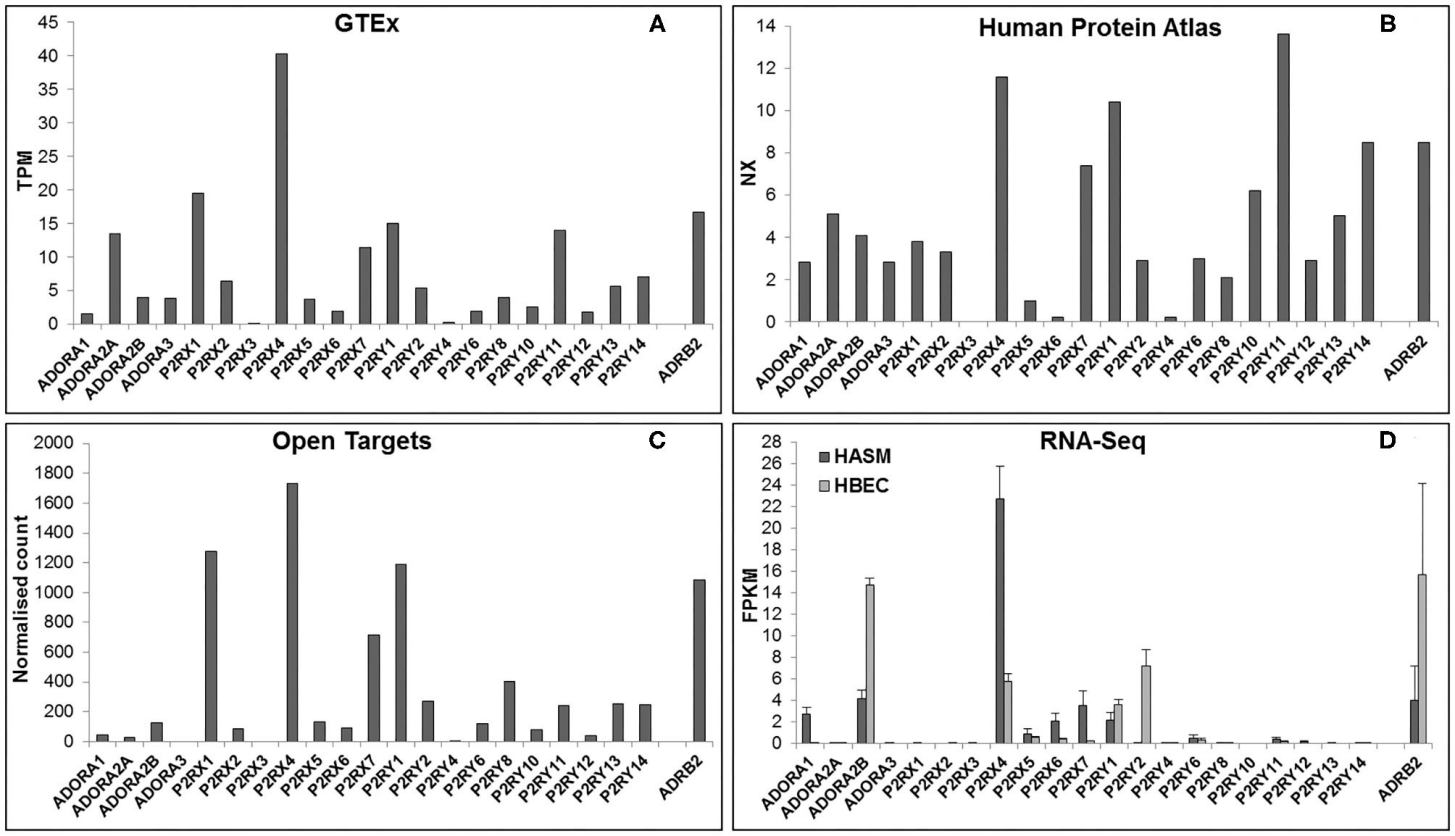
Purinergic receptor RNA expression in lung and airway tissue from multiple online bioinformatics resources and novel RNA-Seq data. As a reference, expression data are also provided for the beta-2 adrenergic receptor (ADRB2), a GPCR that is currently a target for the treatment of respiratory disease. RNA expression data for each gene in lung tissue were gathered from three online resources: **(A)** GTEx Portal {data are median transcripts per million (TPM), *n* = 578 [www.gtexportal.org (v7) (23), date first accessed: 01/04/2020]}, **(B)** Human Protein Atlas {data are consensus normalized expression (NX), *n* = 438 [http://www.proteinatlas.org (v19.3) (24, 25), date first accessed: 06/04/2020]}, and **(C)** Open Targets Platform {data are normalized counts, *n* = 374 [www.targetvalidation.org (v3.18.0) (26), date first accessed: 13/04/2020]}. **(D)** RNA expression data for each purinergic receptor were also gathered from RNA-Seq data on two primary cell types from lung and airway tissue: human airway smooth muscle (HASM; dark gray bars) and human bronchial epithelial cells (HBEC; light gray bars). RNA-Seq data are mean ± SEM fragments per kilobase per million mapped reads (FPKM); *n* = 5 HASM donors and *n* = 8 HBEC donors (27).

In order to be a suitable therapeutic target, a purinergic receptor should be expressed in the target tissue(s) and involved in the pathophysiology of the disease. Fortunately, several bioinformatics resources are freely available online which provide gene expression data at the mRNA and protein levels in a wide range of tissues. Figure 2 depicts RNA expression data for each purinergic receptor in lung tissue obtained from three online bioinformatics resources: GTEx [available from www.gtexportal.org (23)], The Human Protein Atlas [HPA, available from http://www.proteinatlas.org (24, 25)] and Open Targets Platform [available from www.targetvalidation.org (26)]. Figure 2 also shows RNA-Seq data from two primary cell types: human airway smooth muscle (HASM) and human bronchial epithelial cells (HBEC). These datasets were described and utilized in our previous studies (27), and expression data for individual purinergic receptors in each HASM and HBEC donor can be found in the Supplementary Material. These RNA expression data shown in Figure 2 are also listed in Table 2 below, alongside the currently available qualitative protein expression data from lung and airway tissue obtained from The HPA and Open Targets, with higher expression levels indicated by a darker color. To aid interpretation of these results, additional RNA expression data for the beta-2 adrenergic receptor (ADRB2) has also been provided in Table 2 and Figure 2 to provide some context to the therapeutic potential of purinergic receptors, since short- and long-acting agonists of ADRB2 are effective bronchodilators commonly used in the treatment of asthma (28, 29). Unfortunately, there was no protein expression data available for ADRB2 in these datasets.

**Table 2:** Purinergic receptor RNA and protein expression in lung and airway tissue from multiple online bioinformatics resources and novel RNA-Seq data.

	**RNA EXPRESSION in lung/airway tissue**					**PROTEIN EXPRESSION in lung/airway tissue**			
**Purinergic**	**GTEx**	**HPA**	**Open Targets**	**HASM**	**HBEC**	**Open**	**Human Protein Atlas (HPA)**		
**receptor**	**(TPM)**	**(NX)**	**(normalized counts)**	**(FPKM)**	**(FPKM)**	**Targets**			
							**Macrophages**	**Pneumocytes**	**Airway**
									**epithelial cells**
ADORA1	1.566	2.8	44	2.686	0.017	No data	No data		
ADORA2A	13.43	5.1	30	0.003	0.006	Not expressed	Not detected	Not detected	Not detected
ADORA2B	4.007	4.1	126.5	4.156	14.768	No data	No data		
ADORA3	3.892	2.8	No data	0.051	0	No data	No data		
P2RX1	19.5	3.8	1277	0.02	0	No data	No data		
P2RX2	6.349	3.3	84	0	0.002	No data	No data		
P2RX3	0.027	0	No data	0.003	0	No data	No data		
P2RX4	40.36	11.6	1733.5	22.717	5.797	High	High	Not detected	Medium
P2RX5	3.752	1	132.5	0.844	0.557	Not expressed	Not detected	Not detected	Not detected
P2RX6	1.95	0.2	93.5	2.106	0.367	Not expressed	Not detected	Not detected	Not detected
P2RX7	11.39	7.4	715.5	3.536	0.211	Medium	Medium	Not detected	Medium
P2RY1	14.95	10.4	1188.5	2.144	3.584	Medium	Medium	Not detected	Medium
P2RY2	5.423	2.9	273	0.049	7.175	Low	Low	Low	High
P2RY4	0.224	0.2	6	0.013	0.029	No data	No data		
P2RY6	1.925	3	118.5	0.46	0.318	Not expressed	Not detected	Not detected	Not detected
P2RY8	3.935	2.1	404.5	0.035	0.001	Not expressed	Not detected	Not detected	Not detected
P2RY10	2.588	6.2	81	0	0	Not expressed	Not detected	Not detected	Not detected
P2RY11	13.99	13.6	240	0.411	0.153	Medium	Medium	Low	Medium
P2RY12	1.728	2.9	37.5	0.134	0	Not expressed	Not detected	Not detected	Not detected
P2RY13	5.613	5	253	0.002	0	Low	Low	Not detected	Not detected
P2RY14	7.075	8.5	250	0.002	0.003	Medium	Medium	Medium	Medium
ADRB2	16.73	8.5	1085	3.966	15.652	*No data*	*No data*		

*As a reference, expression data are also provided for the beta-2 adrenergic receptor (ADRB2), a GPCR that is currently a target for the treatment of respiratory disease. RNA expression data for each gene in lung tissue were gathered from three online resources: GTEx Portal {data are median transcripts per million (TPM), n = 578 [www.gtexportal.org (v7) (23), date first accessed: 01/04/2020]}, Human Protein Atlas {HPA, data are consensus normalized expression (NX), n = 438 [http://www.proteinatlas.org (v19.3) (24, 25), date first accessed: 06/04/2020]} and Open Targets Platform {data are normalized counts, n = 374 [www.targetvalidation.org (v3.18.0) (26), date first accessed: 13/04/2020]}. RNA expression data for each purinergic receptor were also gathered from RNA-Seq data on two primary cell types from lung and airway tissue: human airway smooth muscle (HASM) and human bronchial epithelial cells (HBEC). RNA-Seq data are mean fragments per kilobase per million mapped reads (FPKM); n = 5 HASM donors and n = 8 HBEC donors (27). Where available, qualitative purinergic receptor protein expression data in lung and airway tissue were gathered from two online resources: Open Targets Platform and Human Protein Atlas. Each dataset is color-coded according to expression level, with darker color indicating higher expression levels*.

### P2X Receptors

Figure 2 and Table 2 show that P2RX1, P2RX4, and P2RX7 RNA are the most highly expressed P2X receptors in all three online bioinformatics resources, suggesting that these receptors are well-expressed in lung and airway tissue. Although protein expression data was not available for every P2X receptor, the existing protein expression data supports the RNA data, as P2RX4 and P2RX7 protein expression in lung and airway tissue was categorized as either “high” or “medium.” However, while P2RX4 RNA was also expressed in both RNA-Seq datasets, P2RX1 expression was not detected in either dataset (FPKM < 1) and P2RX7 RNA was only detected in the HASM dataset. Interestingly, these receptors were expressed at similar levels to ADRB2; therefore suggesting these receptors may have therapeutic potential. P2RX7 most closely resembled ADRB2 expression, except in the HBEC dataset, where ADRB2 was very highly expressed but P2RX7 could not be detected (FPKM < 1). P2RX4 was more highly expressed than ADRB2 in every dataset except the HBEC dataset, although P2RX4 was the only P2X receptor to be expressed in this dataset. Conversely, P2RX2, P2RX5, and P2RX6 are expressed at much lower levels in all datasets analyzed, whereas P2RX3 could not be detected in any dataset (all expression data <1), indicating that these receptors are less likely to be expressed in lung and airway tissue. Again, the available protein expression data supports the RNA data, as P2RX5 and P2RX6 protein expression was not detected. Taken together, these data suggest that of the P2X receptors, P2RX1, P2RX4, and P2RX7 are likely to be the most credible targets for respiratory diseases such as asthma.

### P2Y Receptors

Figure 2 and Table 2 show that P2RY1 RNA was the only P2Y receptor to be highly expressed across all five datasets, while P2RY2 RNA was also expressed at a lower level, except in the HBEC RNA-Seq dataset where its expression was greater than that of P2RY1. This indicates that both receptors are expressed in lung and airway tissue, which is also supported by the protein expression data. Interestingly, the P2RY1 expression levels were very similar to that of ADRB2 in all datasets, with the exception of the HBEC dataset where ADRB2 expression was very high, although P2RY1 was also moderately expressed in this dataset. This suggests that targeting P2RY1 in particular may have therapeutic potential in the lung. Furthermore, P2RY2 was the most highly expressed P2Y receptor in the HBEC dataset, but its expression levels are still lower than that of ADRB2. Although no protein data was available, P2RY4 RNA expression was not expressed in any dataset, suggesting that this receptor is not expressed in lung and airway tissue. However, there was some variability in expression patterns of the remaining P2Y receptors across the datasets, with no expression detected for any of these receptors in the HASM or HBEC RNA-Seq datasets (FPKM < 1), although expression was detected in the online bioinformatics datasets. P2RY11 RNA expression was at a similar level to that of P2RX7 and P2RY1 in the GTEx and HPA (but not Open Targets) datasets, indicating that P2RY11 expression in lung and airway tissue is at a similar level to those receptors. This is supported by the protein expression data, with P2RY11 protein expression similarly categorized as “medium.” Furthermore, RNA expression for P2RY13 and P2RY14 is comparable to that of P2RY2 in the GTEx and Open Targets datasets, although the HPA dataset shows their expression to be greater than that of P2RY2. These data suggest that expression of P2RY13 and P2RY14 in lung and airway tissue is similar to that of P2RY2. This is also supported by the protein expression data, as expression for P2RY13 and P2RY14 was categorized as “low” and “medium,” respectively. Generally, RNA expression levels for P2RY6, P2RY8, P2RY10, and P2RY12 were lower than that of P2RY2 across all three online resources, with the exception of P2RY10, which was shown to be well-expressed in the HPA dataset. Together, these data indicate that these receptors are the least well-expressed P2Y receptors in lung and airway tissue, and this is supported by the protein expression data, since no protein was detected for any of these receptors.

### P1 (Adenosine) Receptors

The expression patterns of the P1 family of receptors were more variable between the datasets investigated (see Figure 2 and Table 2). While the GTEx and HPA datasets show consistent but low-level RNA expression for all four P1 receptor subtypes, the other datasets indicate a much lower expression level, with the exception of ADORA2A in the GTEx dataset, ADORA1 in the HASM RNA-Seq dataset, and ADORA2B in the HASM and HBEC RNA-Seq datasets. Generally, these data suggest that there is low level expression of P1 receptors in lung and airway tissue. However, the particularly high expression levels of ADORA2B in the HBEC and HASM RNA-Seq datasets suggest that expression may be higher in some cell types. Interestingly, ADORA2B expression in the RNA-Seq datasets was very similar to that of ADRB2; therefore suggesting that ADORA2B may have therapeutic potential in these particular cell types. However, ADORA2B expression was much lower than that of ADRB2 in all three online resources. Unfortunately, protein expression data is lacking for the majority of P1 receptors, but ADORA2A protein was not detected.

### Expression Data at the Single Cell Level Reveal That, Within Lung Tissue, Purinergic Receptors Tend to Be Highly Expressed in Immune Cells and Moderately Expressed in Epithelial Cells

While it is valuable to use online resources, such as the HPA Tissue Atlas, to determine gene expression at the tissue level, it is also beneficial to know in which specific cell types the gene of interest is expressed to allow for better selection of potential therapeutic targets and for better understanding of the biological mechanisms involved. Fortunately, the HPA has recently developed a Cell Type Atlas, which provides gene expression data for the various cell types within different tissues [available from http://www.proteinatlas.org (24, 25)]. The single cell RNA-Seq data from lung tissue reveals 11 different clusters of cell types, which include epithelial cells (alveolar type 2, alveolar type 1, ciliated and club cells), blood and immune cells (macrophages, T cells and granulocytes), endothelial cells and fibroblasts. Interestingly, two distinct clusters exist for alveolar type 2 cells and macrophages, each with slightly different expression levels of their respective cell type-specific markers, indicating different sub-populations of both cell types. The purinergic receptor gene expression data in each cell type cluster is presented in Table 3 below, with higher expression levels indicated by a darker color.

**Table 3:** Purinergic receptor expression in different cell types within lung tissue.

	**Cluster-0**	**Cluster-2**	**Cluster-3**	**Cluster-4**	**Cluster-5**	**Cluster-9**	**Cluster-1**	**Cluster-6**	**Cluster-7**	**Cluster-8**	**Cluster-10**
	***n* = 1,023**	***n* = 613**	***n* = 518**	***n* = 321**	***n* = 296**	***n* = 182**	***n* = 750**	***n* = 275**	***n* = 245**	***n* = 207**	***n* = 169**
	**Macrophages**	**Macrophages**	**T-cells**	**Granulocytes**	**Fibroblasts**	**Endothelial**	**Alveolar type 2**	**Alveolar type 2**	**Club**	**Ciliated**	**Alveolar type 1**
ADORA1											
ADORA2A						5.1	0.8				
ADORA2B	4.2		2.1		4		5.8	3.5	11.6	12.4	5.4
ADORA3	0.6	1.3	2.1	6.1							
P2RX1	24.8	43.2	24.7	285.7	6.7	5.1	1.6	17.5	3.3	2.1	5.4
P2RX2											
P2RX3											
P2RX4	72.6	35.3	14.4	33.4	9.4	20.6	20.6	35.1	21.6	14.4	10.9
P2RX5	0.6	1.3	4.1		1.3			3.5			
P2RX6				6.1				3.5		6.2	
P2RX7	46	55	24.7	15.2	16.1	36	5.8	7	10	10.3	5.4
P2RY1	19.4	6.5	4.1	24.3	4		0.8				
P2RY2		5.2					0.8		6.6		5.4
P2RY4											
P2RY6	3	11.8	8.2		2.7			3.5		2.1	
P2RY8	0.6	7.9	16.5	3		10.3	0.8	7	1.7		
P2RY10		3.9	55.5	6.1	1.3		1.6	3.5	1.7		
P2RY11	13.9	3.9	10.3	9.1			4.9	3.5	3.3	4.1	10.9
P2RY12	1.8	1.3		3							
P2RY13	59.9	81.1	12.3	6.1	5.4		1.6	28.1		2.1	
P2RY14	3.6	36.6	20.6	45.6			0.8			2.1	5.4
ADRB2	24.2	11.8	35	124.6	10.7	30.8	31.3	66.7	48.2	12.4	190.2

*As a reference, expression data are also provided for the beta-2 adrenergic receptor (ADRB2), a GPCR that is currently a target for the treatment of respiratory disease. RNA expression data for each gene in all 11 cell clusters within lung tissue were obtained from the Human Protein Atlas (HPA) Cell Type Atlas [https://www.proteinatlas.org (v20.0) (24, 25), date first accessed: 15/01/2021]. Data are transcripts per million protein coding genes (pTPM), and cell numbers for each individual cell cluster are given at the top of each corresponding column. Data are grouped into broad cell types, with blood and immune cell types grouped in the first four columns, and epithelial cell types grouped in the last five columns. All data are color-coded according to expression level, with darker color indicating higher expression*.

The cell type-specific expression data in Table 3 reflect that of the lung tissue expression data in Table 2, whereby the same purinergic receptors are identified as highly expressed (namely P2RX1, P2RX4, P2RX7, P2RY1, P2Y11, P2Y13, and P2Y14). Interestingly, these receptors are also expressed in multiple cell types. Similarly, those receptors expressed at lower levels or not expressed at all at the lung tissue level are also poorly expressed in the single cell data, and tend to be expressed in fewer cell types. The cell type-specific data also reveal that purinergic receptors are more highly expressed in blood and immune cell types (see the first four columns of Table 3) compared to the other cell types, with P2RX1, P2RX4, P2RX7, and P2RY13 expressed at particularly high levels. However, P2RX4 is also reasonably well-expressed across all epithelial cell types; while ADORA2B, P2RX7, and P2RY11 are moderately expressed in epithelial cells (see the last five columns of Table 3), while P2RX4 and P2RX7 are also well-expressed in endothelial cells and fibroblasts.

### The IPF Cell Atlas Allows for Comparison of Gene Expression Between Healthy and Diseased Lung Tissue

The GTEx, Human Protein Atlas and Open Targets resources provide useful bioinformatics data on gene expression levels within healthy tissues. However, it would be beneficial to also have similar resources for diseased tissues, since gene expression can often be altered in pathological conditions. As yet, there is no available open access resource that examines gene expression at the single cell level in asthmatic tissues. Nevertheless, the Idiopathic Pulmonary Fibrosis (IPF) Cell Atlas is an online resource which provides visual outputs of gene expression data at the single cell level from both normal and IPF lung tissue (available from https://p2med.shinyapps.io/IPFCellAtlas/), and can therefore be used to give an indication of purinergic receptor expression at the single cell level in healthy and diseased lung tissue. The IPF Cell Atlas allows the user to visualize expression levels of their gene of interest in each of the four available datasets in two broad representations, either as a Uniform Manifold Approximation and Projection (UMAP) plot in three metrics (gene expression, disease, and cell type), or as a violin, bar, density or box plot of gene expression in each of the different cell types, which can then be broken down further into disease type.

**Figure 3:**
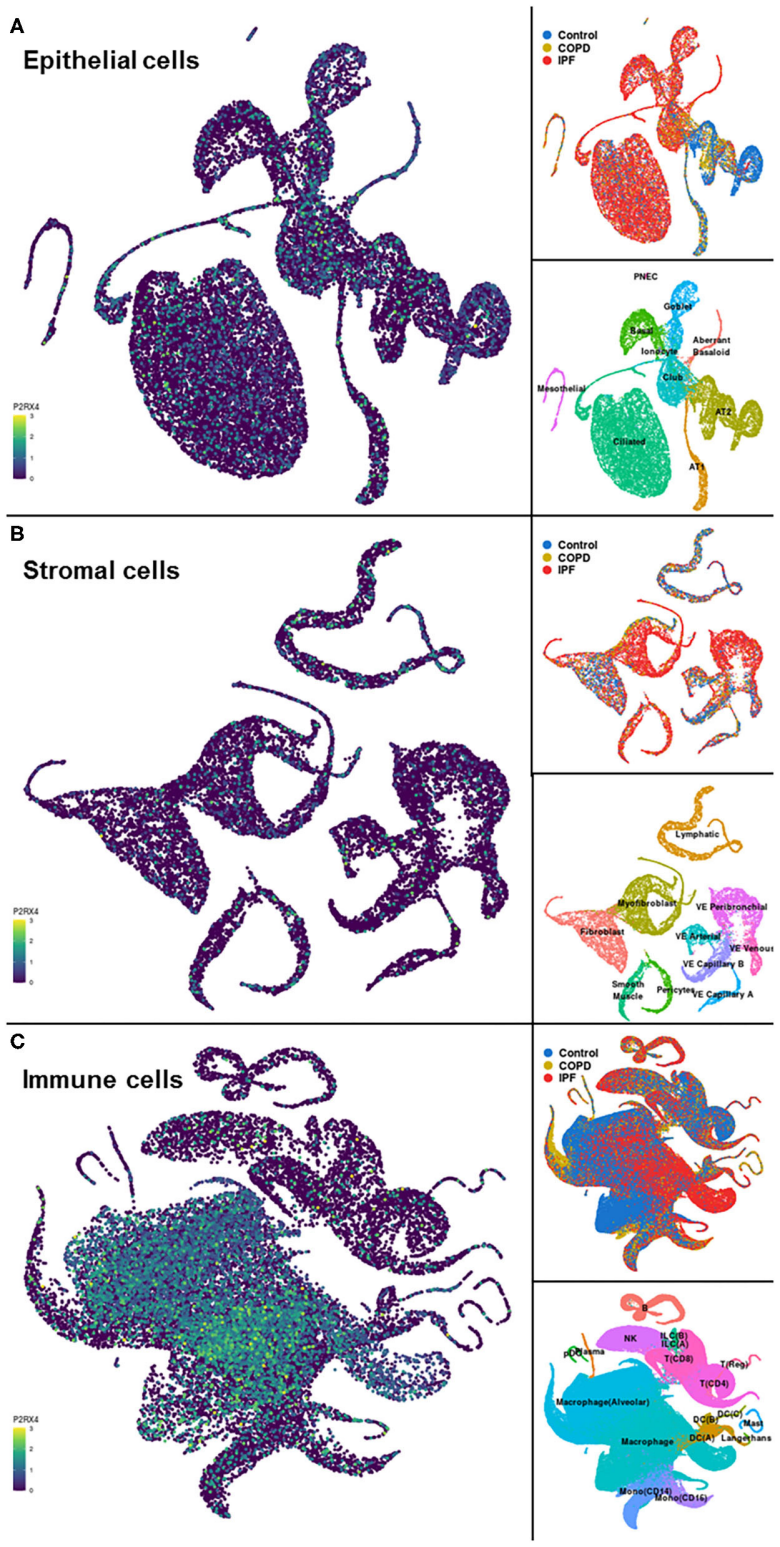
Visualizations of cell type-specific distribution of P2RX4 expression within healthy and diseased lung tissue. Left-hand panels show Uniform Manifold Approximation and Projection (UMAP) plots of P2RX4 gene expression levels in epithelial **(A)**, stromal **(B)**, and immune **(C)** cell types, with lighter colors indicating higher expression. Upper and lower right-hand panels show disease state and cell type associated with each region of the corresponding UMAP plot, respectively. Data are *n* = 312,928 cells from 29 control, 18 chronic obstructive pulmonary disease (COPD) and 32 idiopathic pulmonary fibrosis (IPF) lungs. Images are from the “UMAP Explorer” tool in the IPF Cell Atlas using the Kaminski/Rosas dataset [https://p2med.shinyapps.io/IPFCellAtlas/, date first accessed: 22/04/2020 (30, 31)].

**Figure 4:**
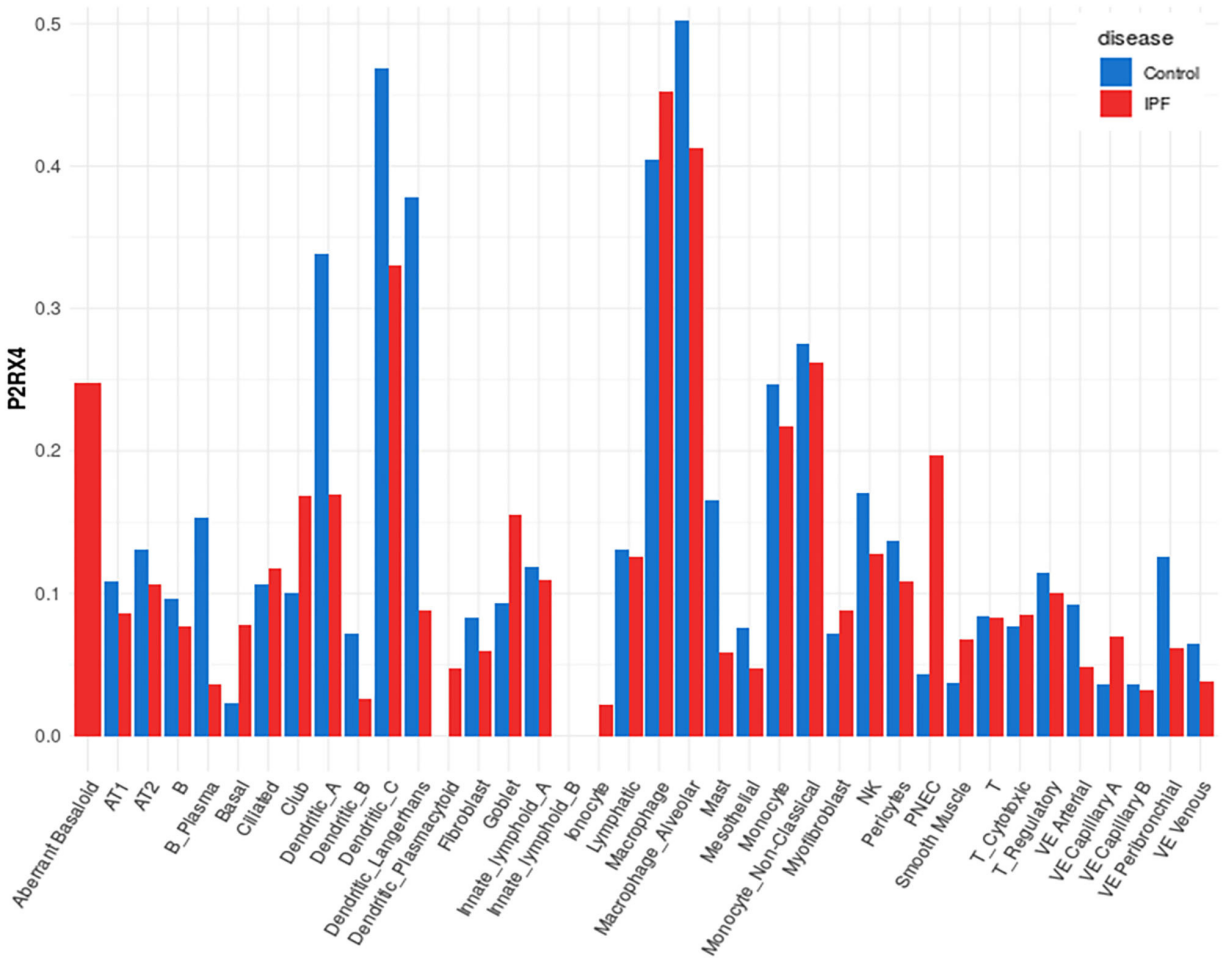
Plot of cell type-specific distribution of P2RX4 expression within healthy and diseased lung tissue. Bar plot showing average P2RX4 gene expression in different cell types of lung tissue, further broken down into disease state, with expression levels from healthy control lung tissue shown in blue bars (*n* = 29) and idiopathic pulmonary fibrosis (IPF) lung tissue in red bars (*n* = 32). Image is from the “Gene Explorer” tool in the IPF Cell Atlas using the Kaminski/Rosas dataset [https://p2med.shinyapps.io/IPFCellAtlas/, date first accessed: 22/04/2020 (30, 31)].

A representative example of data obtainable from the IPF Cell Atlas is shown below, with P2RX4 expression shown across the different cell and disease types from the Kaminski/Rosas dataset (30, 31) represented as UMAP plots in Figure 3, and as a gene expression bar plot broken down by disease type in Figure 4. P2RX4 was selected for these representative figures because it was shown to be very highly expressed across all datasets investigated (see Table 2 and Figure 2). The left-hand panel of each UMAP plot in Figure 3 shows gene expression level, with lighter colors indicating higher expression, and the upper and the lower right-hand panels of each plot indicate the disease state and cell type associated with each region of the plot, respectively. These figures can both be used to demonstrate that P2RX4 is highly expressed in macrophages and alveolar macrophages, and moderately expressed in several epithelial cell types, suggesting that P2RX4 could be a potential therapeutic target for these cell types. Conversely, these figures both show low expression levels in stromal cell types, indicating that that P2RX4 is an unlikely therapeutic target for these cell types. Figure 4 can also be used to show that P2RX4 may not be a useful therapeutic target for IPF, since its expression levels are generally quite similar between healthy and IPF tissue.

**Figure 5:**
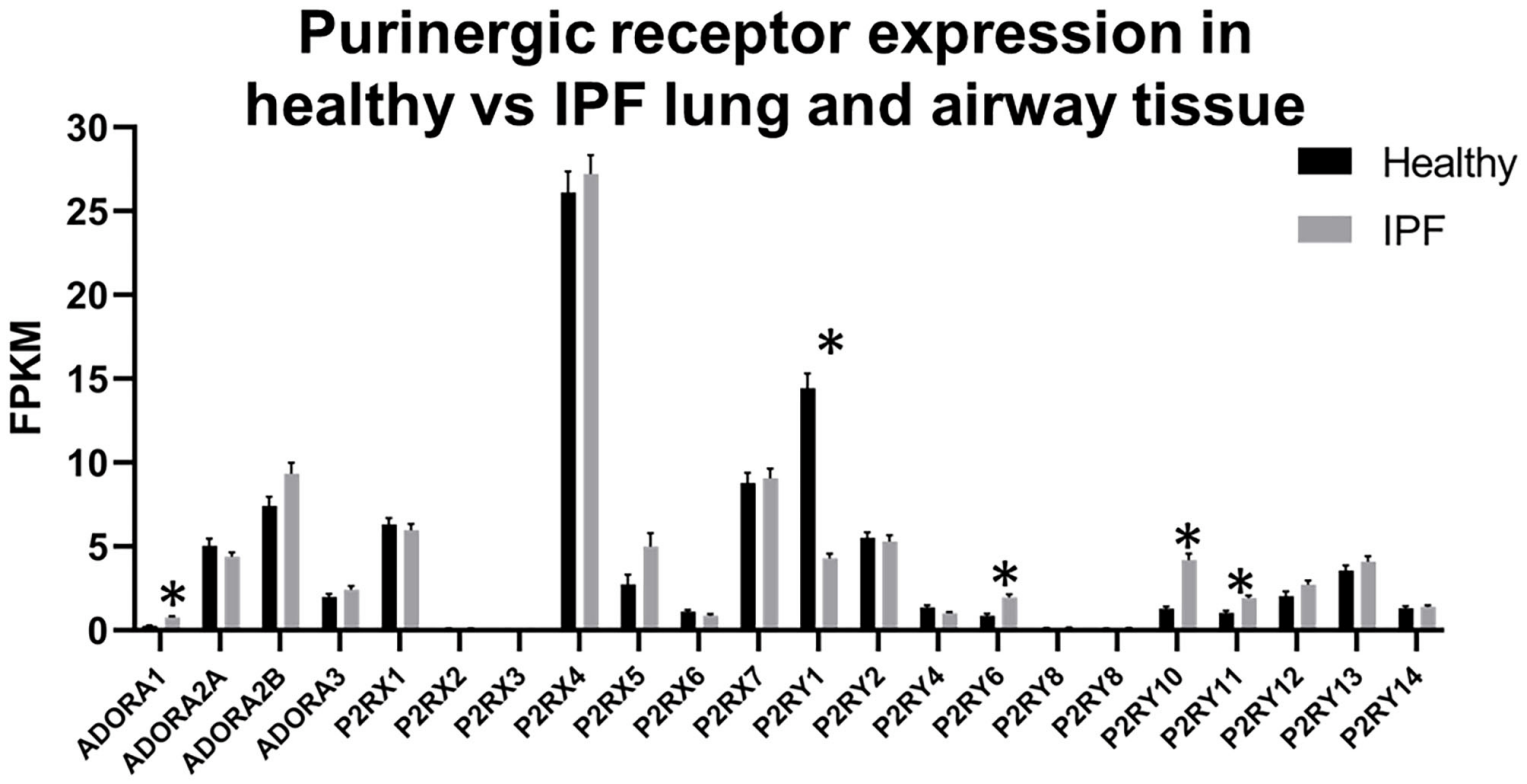
Purinergic receptor expression in healthy vs. idiopathic pulmonary fibrosis (IPF) lung tissue. Data are mean fragments per kilobase per million mapped reads (FPKM) for each purinergic receptor from healthy control (black bars, *n* = 35) and IPF (gray bars, *n* = 49) lung tissue [data accessible at NCBI GEO database (32), accession GSE124685 (31)]. Multiple *t*-tests, correcting for multiple comparisons using the Holm-Sidak method, was performed using GraphPad Prism, v.8.4.3 (^*^p < 0.001).

While the IPF Cell Atlas provides useful visualizations of gene expression across different cell types in healthy and IPF lung tissue, it can be difficult to accurately identify potential therapeutic targets without numerical data. Fortunately, it is possible to access numerical data used for each of the IPF Cell Atlas datasets, as there are links to the raw data which have been uploaded to the Gene Expression Omnibus (GEO), an online repository for functional genomics data [https://www.ncbi.nlm.nih.gov/geo/ (32)]. As shown in Figure 5 below, when data for all cell types are considered together, purinergic receptors are generally expressed at similar levels in healthy and IPF tissue [data accessible at NCBI GEO database (32), accession GSE124685 (31)]. However, P2RY1 expression is significantly decreased by 3-fold in IPF tissue compared to normal (4.286 ± 0.277 vs. 14.431 ± 0.872), whereas there is significantly increased expression of ADORA1 (3-fold; 0.759 ± 0.066 vs. 0.257 ± 0.033), P2RY6 (2-fold; 1.967 ± 0.168 vs. 0.853 ± 0.125), P2RY10 (3-fold; 4.195 ± 0.362 vs. 1.286 ± 0.126), and P2RY11 (2-fold; 1.902 ± 0.150 vs. 1.027 ± 0.127) in IPF tissue compared to normal (*p* < 0.001), therefore suggesting that these could be potential therapeutic targets for IPF.

### Involvement of Purinergic Receptors in the Pathophysiology of Lung Diseases, Including Asthma

Many purinergic receptors have been shown to be expressed either at the mRNA or protein level by different cell types within the lung, and by immune cells which can be recruited to the lungs, particularly under disease conditions (33–35). Not only is purinergic signaling important for many aspects of lung physiology, but aberrant purinergic signaling has been associated with numerous lung pathologies, thus revealing potential therapeutic targets (2, 3). As would be expected given the bioinformatics data presented above, a review of the literature also indicates that purinergic receptors can play an important role in immune cells, for example in host defense. They are also implicated in inflammatory lung diseases, including asthma (see Table 4 for a summary of the evidence showing involvement of purinergic receptors in asthma alongside the expression data from the bioinformatics analysis). However, the precise roles of purinergic receptors within inflammation signaling are still to be fully elucidated. At high levels, extracellular ATP acts as a “danger signal” to recruit and activate immune cells, often by stimulating P2 receptors (34). Conversely, adenosine usually has anti-inflammatory roles through its actions on P1 receptors, but under chronically increased adenosine levels, P1 receptor stimulation exerts a pro-inflammatory response which can lead to lung disease development and progression (57).

**Table 4:** Summary of expression data from bioinformatics resources and evidence for the role(s) in asthma pathophysiology from the literature for each purinergic receptor.

**Purinergic receptor**	**Summary of expression data**	**Evidence for role(s) in asthma**
ADORA1	Weak in most datasets	Increased expression in asthma, activation causes bronchoconstriction and mucus production (36) Implicated in human asthma susceptibility by GWAS (17)
ADORA2A	Moderate in some datasets	Controls immune cell balance by increasing immunosuppressive Tregs and decreasing pro-inflammatory Th17 cells (37)
ADORA2B	Strong in some datasets	Induces inflammation and bronchial hyper-responsiveness (38) Combined ADORA2B and glucocorticoid agonism induces anti-inflammatory gene transcription (39)
ADORA3	Weak in most datasets	Role not yet fully understood (40)
P2RX1	Strong in most datasets	Involved in eosinophil adhesion—impaired in asthmatics (41)
P2RX2	Weak in most datasets	No information on role in asthma
P2RX3	None in any dataset	No information on role in asthma
P2RX4	Strong in all datasets	Involved in immune cell infiltration, collagen deposition, goblet cell hyperplasia, mucus production and phenotype switching on bronchial smooth muscle cells (42–44)
P2RX5	Weak in all datasets	No information on role in asthma
P2RX6	Weak in all datasets	No information on role in asthma
P2RX7	Strong in most datasets	Histamine release from human lung mast cells (7) Activates NLRP3 inflammasome in dendritic cells, promoting airway inflammation and hyper-responsiveness (10) Promotes polarization of alveolar macrophages to pro-inflammatory subtype (11)
P2RY1	Strong in all datasets	Drives pulmonary leukocyte recruitment after allergen challenge (45)
P2RY2	Moderate in most datasets	Mediates pro-allergic Th2 response to airborne allergens (46) Induces chemotaxis and accumulation of dendritic cells and eosinophils and reactive oxygen species production (47, 48)
P2RY4	None in any dataset	No information on role in asthma
P2RY6	Weak in all datasets	Involved in airway remodeling, release of pro-inflammatory cytokine and chemokines, and migration of mast cells (49, 50) Inhibits inflammatory responses (12, 51)
P2RY8	Weak in all datasets	No information on role in asthma
P2RY10	Weak in all datasets	No information on role in asthma
P2RY11	Strong in most datasets	No information on role in asthma
P2RY12	Weak in all datasets	Required to mediate pro-inflammatory effects of leukotriene E4 (52) Increased expression after allergen challenge, and involved in airway hyper-responsiveness and infiltration of immune cells, including eosinophils (53) Role in human lung function (54)
P2RY13	Moderate in most datasets	Associated with asthma by human GWAS, increased expression in mouse epithelia, eosinophils and neutrophils after allergen challenge, and induced IL-33 release and eosinophil infiltration into lung (55)
P2RY14	Strong in most datasets	Associated with asthma by human GWAS, increased expression in mouse epithelia, eosinophils and neutrophils after allergen challenge, and induced IL-33 release and eosinophil infiltration into lung (55) Role in promoting human mast cell degranulation (56)

*Unless otherwise stated, the literature is from murine allergic lung inflammation as a model for asthma*.

Both ATP itself and a range of P2 receptors have been shown to play critical roles in pulmonary inflammation, and so should be considered potential therapeutic targets for inflammatory lung diseases, particularly asthma and COPD (58). Aerosolized ATP exacerbates symptoms in both asthmatics (59) and COPD patients (60), and ATP is also involved in smoke-induced lung inflammation in mice via activation of P2RX7 and P2RY2 (61, 62). Furthermore, P2RY2 is involved in mediating murine lung allergic inflammation via IL-33 release and stimulation of Th2 response pathways (46). This results in chemotaxis and accumulation of dendritic cells and eosinophils and production of reactive oxygen species (47, 48). P2RX7 also has roles in several different immune cell types. For example, P2RX7 activation has been shown to induce human lung mast cells to release histamine (7), polarize alveolar macrophages toward a pro-inflammatory subtype in asthmatic mice (11) and activate the NLRP3 inflammasome in dendritic cells, thus promoting airway inflammation and hyper-responsiveness in asthmatic mice (10). P2RX1 has been shown to be involved in integrin-dependent adhesion of healthy human eosinophils, but this is impaired in asthmatic eosinophils as a likely result of the increased concentration of extracellular nucleotides present in asthmatic lungs (41). P2RX4 activation has also been shown to contribute to inflammation and airway remodeling in a mouse model of allergic asthma by inducing immune cell infiltration, collagen deposition, goblet cell hyperplasia and mucus production, as well as causing phenotype switching of bronchial smooth muscle cells (42–44).

In allergic mouse models of lung inflammation, activation of P2RY1 expressed on platelets has been shown to drive pulmonary leukocyte recruitment via RhoA signaling following allergen challenge (45). P2RY12 is required to mediate the pro-inflammatory effects of leukotriene E4, a potent stimulator of airway hyper-responsiveness and mucosal eosinophilia in human asthmatics (52). P2RY12 has also been implicated in modulating human lung function from genetic studies looking at gene environment interactions (54). Furthermore, P2RY12 expression in mouse lung is increased following allergen challenge, and is involved in hyper-responsiveness and immune cell infiltration in the airways (53). Both P2RY13 and P2RY14 have been associated with asthma by GWAS analysis, while studies in mice showed expression of both receptors is increased following allergen challenge, particularly in airway epithelia, neutrophils and eosinophils, and both receptors are involved in IL-33 release and infiltration of eosinophils into the lung (55). P2RY14 has also been shown to promote degranulation in the human mast cell line, LAD2 (56). However, there are differences in the literature regarding the effects of P2RY6 activation in mouse models of allergic airway inflammation. Some studies indicate a pro-inflammatory effect, with P2RY6 shown to induce airway inflammation and remodeling via the release of pro-inflammatory cytokines and chemokines from epithelia and mast cells (49, 50). Conversely, other studies suggest an anti-inflammatory effect, with P2RY6 shown to inhibit allergen-induced inflammatory responses and airway remodeling (12, 51).

Similarly, adenosine and P1 receptors are also considered potential therapeutic targets for inflammatory lung diseases, given their involvement in pulmonary inflammation. Adenosine is known to induce bronchoconstriction in patients with asthma (63) and COPD (64), but this effect is not seen in healthy individuals. Furthermore, increased levels of adenosine have been found in bronchoalveolar lavage fluid (BALF) and exhaled breath condensates of patients with asthma (65, 66) and COPD (67, 68). All four P1 receptors have been implicated in both diseases; therefore they have received much interest as potential therapeutic targets. ADORA1 expression is increased in asthmatic patients as well as in animal models of allergic airway inflammation, and ADORA1 activation results in bronchoconstriction and mucus production. Thus, ADORA1 is thought to be involved in airway inflammation and remodeling in chronic lung diseases such as asthma and COPD (35). ADORA1 has also been implicated in asthma susceptibility by GWAS and functional studies (17). Interestingly, an ADORA1 antagonist was shown to decrease the allergic response to house dust mites in a rabbit model of allergic asthma (36). Conversely, ADORA2A agonists are considered to be potential treatments for asthma and COPD, since ADORA2A is important for controlling the balance between the immune cells in allergic asthma by simultaneously increasing immunosuppressive Treg cells and decreasing pro-inflammatory Th17 cells in mice (37). Although ADORA2A agonists have shown promising results in animal models of asthma and COPD, there has been less success in human clinical trials, largely due to limited efficacy (35). Selective ADORA2B antagonists have demonstrated anti-inflammatory effects in an allergic asthma mouse model (38), whereas combined agonism of ADORA2B and glucocorticoid receptors has been shown to induce transcription of anti-inflammatory genes in BEAS-2B cells, a human airway epithelial cell line (39). However, the role of ADORA3 in human airway inflammation is not yet fully understood, thus it is still unclear whether agonists or antagonists of ADORA3 would be of any value for the treatment of asthma and COPD (40).

### Clinical Studies Targeting Purinergic Receptors in Other Respiratory Diseases

There has also been much interest in targeting purinergic receptors for other respiratory diseases. For example, gefapixant (formerly known as AF-219 and MK-7264), a negative allosteric modulator of P2RX3 and P2RX2/3, has been extensively investigated for the treatment of refractory chronic cough (58, 69). Phase I and phase II clinical trials have all demonstrated good safety and efficacy of gefapixant as an antitussive even at lower dosages of between 30 and 50 mg (twice daily). However, reduced or total loss of taste was a common adverse event in all trials, although the frequency of taste disturbance was reduced with lower doses (70–72). Phase III clinical trials are currently underway, with over 2,000 patients randomized to placebo, 15 or 45 mg of gefapixant twice-daily for up to 12 months. Cough frequency and severity are being measured as efficacy outcomes, and adverse events experienced are also being recorded (73). In an attempt to address the taste disturbances commonly associated with gefapixant, phase II clinical trials are also currently in progress for three other antagonists that are more specific to P2RX3: BLU-5937 (Bellus), BAY 1817080 (Bayer), and S-600918 (Shionogi). While all three compounds have been shown to have antitussive effects, taste disturbance is still experienced with these compounds, albeit to a lesser extent than with gefapixant (69).

Given their ability to alter ion transport (see Figure 1), purinergic receptors are also considered promising targets for the treatment of cystic fibrosis (CF), where defective ion transport and subsequent abnormal airway surface liquid volume is caused by mutations in the cystic fibrosis transmembrane regulator (CFTR) gene (2, 3). For example, P2RX4 has been shown to play a critical role in alveolar fluid transport (74, 75), while ADORA1 and ADORA2A are involved in ion transport in CF airway epithelial cells (3). Furthermore, P2RY2 is able to improve mucosal hydration and mucociliary clearance by stimulating ciliary beat frequency and chloride secretion, as well as inhibiting sodium transport, in both normal and CF airway epithelia. Consequently, the safety and efficacy of denufosol tetrasodium (a selective P2RY2 agonist) as a treatment for CF has been investigated in clinical trials. Although denufosol was well-tolerated and showed some evidence of improved lung function in patients with mild CF, there was limited clinical efficacy and the project was ended (76, 77).

## Discussion and Conclusions

In this review, we have demonstrated how bioinformatics resources can be utilized to explore purinergic receptor gene expression in tissues and cell types of interest and to identify potential therapeutic targets. Expression levels of the 21 purinergic receptors in healthy lung and airway tissue were investigated using three online bioinformatics resources (GTEx, HPA and Open Targets), as well as two published RNA-Seq datasets from HASM and HBEC cells. Using these resources, several purinergic receptors were shown to be expressed in healthy lung and airway tissue (see Figure 2 and Table 2). Most notably, P2RX1, P2RX4, P2RX7, P2RY1, P2RY11, and P2RY14 are well-expressed across the datasets, whereas other receptors are poorly expressed, particularly P2RX3 and P2RY4 which could not be detected. Interestingly, the receptor expression data reflects the evidence for the roles of purinergic receptors within asthma in the literature (see Table 4). With the exception of P2RY11, all the well-expressed receptors have been previously implicated in asthma, therefore supporting the therapeutic potential for targeting these receptors. Similarly, receptors with little or no expression do not have evidence of a role within asthma, with the exception of P2RY6 and P2RY12. Furthermore, the recent addition of the HPA Single Cell Atlas has enabled the investigation of gene expression within specific cell types within the tissue of interest. This revealed that purinergic receptors are more highly expressed in immune cell types, and moderately expressed in epithelial cell types (see Table 3). Interestingly, many of the known roles of purinergic receptors in asthma involve immune cells widely understood to be important for the pathogenesis of asthma, including dendritic cells, alveolar macrophages, mast cells and eosinophils (see Table 4 for a summary of roles for purinergic receptors in asthma pathogenesis). This subsequently strengthens the potential for these receptors as effective therapeutic targets for asthma.

While Figure 2 and Table 2 demonstrate that the majority of purinergic receptors are expressed to some extent in lung and airway tissue, they also show discrepancy between the datasets, especially between the online resources and RNA-Seq data. A likely reason for such discrepancy is that the online resources use whole lung tissue, which contains an assortment of cell types. As demonstrated in Table 3, these cell types express purinergic receptors to different extents, which will in turn affect global expression levels within the heterogeneous whole lung tissue. Conversely, the RNA-Seq data are from single cell types, hence purinergic receptor expression will be at a similar level across the cell population. This is particularly evident for the HBEC dataset, in which ADORA2B expression is much greater than that of the other datasets. However, HBECs are epithelial cells, which were shown to express ADORA2B at higher levels than other cell types (Table 3), which could explain these findings. One important issue to note is that only undifferentiated basal HBECs were used for the RNA-Seq studies, so it is possible that there may be some differences in purinergic receptor expression levels in differentiated HBECs.

Furthermore, as the data in Figure 2 and Tables 2 and 3 are from healthy lung tissue, it is likely that the number of immune cells in these tissues would be relatively low compared to that of inflamed lung tissue. Consequently, it is plausible that purinergic receptor expression is different under inflammatory conditions, such as in patients with asthma or COPD, due to the increased number of immune cells expressing the purinergic receptors and potentially altered receptor expression levels in diseased tissues. Therefore, in order to better identify potential therapeutic targets, it may be beneficial to identify changes in purinergic receptor expression under disease conditions. At present, there are no open access single cell lung datasets derived from asthmatic patients, although these should become available in the near future. Despite this, there is evidence in the literature describing altered expression of purinergic receptors in asthma, particularly upregulation of P2RX7 (78), P2RY2 (47), and P2RY6 (49) in asthmatic tissues, therefore identifying these as potential therapeutic targets for asthma. However, useful data can be found from the IPF Cell Atlas, which can be utilized to compare gene expression between control and IPF tissue, albeit in a purely qualitative manner (see Figures 3 and 4). Some of the data used in this resource can be accessed from the GEO data repository, which can then be used to quantitatively compare gene expression in control and IPF tissue. As shown in Figure 5, five purinergic receptors are differentially expressed between control and IPF lung tissue, with decreased P2RY1 expression in IPF tissue and increased expression of ADORA1, P2RY6, P2RY11, and P2RY12 in IPF tissue. This implies that these purinergic receptors may be involved in IPF pathophysiology and may be potential therapeutic targets.

Interestingly, P2RY6 has been previously implicated in IPF pathogenesis, with increased P2RY6 expression observed in lung structural cells from IPF patients and from the mouse model of bleomycin-induced pulmonary fibrosis. Furthermore, P2RY6 activation by UDP was shown to increase human and mouse lung fibroblast proliferation and IL-6 production, whereas fibrosis and inflammation was reduced in P2RY6-deficient mice and in mice treated with a P2RY6 antagonist (79). This supports the data from the IPF Cell Atlas which indicates that P2RY6 may be a potential therapeutic target for IPF. Similar results were also observed for P2RY2 by the same authors (80); therefore suggesting that P2RY2 may also be a potential therapeutic target for IPF. However, P2RY2 levels were unchanged in the IPF Cell Atlas data [see Figure 5 (31)], which perhaps de-prioritizes P2RY2 as a suitable target in human lung disease. This could be because purinergic receptors may have divergent functions between the two species due to their different physiologies. This would result in animal model data which cannot be replicated in humans, since animal models may have different phenotypes compared to humans, or there may be no observable effect in the model at all (34, 81). Unfortunately the literature is full of agents showing efficacy at a range of targets in animal models of respiratory diseases which have subsequently failed to show efficacy in clinical trials in humans.

These species differences when using animal models may also partially explain the limited success in clinical trials of treatments targeting purinergic receptors (34, 35). In addition, lack of potent selective compounds has limited the preclinical and clinical study of purinergic receptor targets. Consequently, it is likely that using more humanized approaches to identify potential therapeutic targets may lead to a better success rate in clinical trials. Bioinformatics approaches, such as those discussed in this review, may help address this issue, since human gene expression data can be obtained at the single cell level, as well as the whole tissue, which can also suggest potential functions for the gene. For example, the purinergic receptors highly expressed on immune cells are likely to be important for lung inflammation. Usually, online bioinformatics resources, such as GTEx, use healthy tissue, but it is arguably more beneficial to understand how gene expression is altered in disease in order to better identify potential therapeutic targets. Although online resources like the IPF Cell Atlas are not yet available for other respiratory diseases, bioinformatics datasets such as RNA-Seq data for diseases such as asthma and COPD are available and can be utilized to identify gene expression changes in these diseases to identify potential therapeutic targets. Such datasets can be accessed via GEO or via the Gateway project facilitated in part by BREATHE, the Health Data Research UK (HDRUK) Hub for Respiratory Health.

In summary, purinergic receptors are abundantly expressed within lung and airway tissue, particularly within immune cells, and have been demonstrated to play critical roles in lung physiology and pathophysiology (as summarized in Table 4). Therefore, these receptors are likely to make promising therapeutic targets for respiratory diseases, including asthma. However, it is crucial to identify the specific receptors concerned and have a greater understanding of their involvement in the disease mechanism in order to develop successful therapeutics. To this end, bioinformatics datasets are a valuable tool by which potential therapeutic targets can be identified based upon their expression levels in healthy and diseased tissue.

## Author Contributions

All authors listed have made a substantial, direct and intellectual contribution to the work, and approved it for publication.

## Conflict of Interest

The authors declare that the research was conducted in the absence of any commercial or financial relationships that could be construed as a potential conflict of interest.
