# Supplementary material for: Purinergic Receptors in the Airways: Potential Therapeutic Targets for Asthma?
Source: Front Allergy. 2021 May 31;2:677677. doi: 10.3389/falgy.2021.677677 (PMC8974712; doi:10.3389/falgy.2021.677677)
Supplement: Supplementary file 1 [file Data_Sheet_1.DOCX]

# Supplementary Material – Purinergic receptor gene expression from RNA-Seq data

The following supplementary data provide the RNA-Seq studies performed in (Hall et al., 2021).

|  | **HASM (FPKM)** | | | | |
| --- | --- | --- | --- | --- | --- |
| **Receptor** | **Donor 1** | **Donor 2** | **Donor 3** | **Donor 4** | **Donor 5** |
| ADORA1 | 4.99158 | 1.82065 | 0.986621 | 3.51196 | 2.12075 |
| ADORA2A | 0 | 0 | 0 | 0 | 0.01643 |
| ADORA2B | 3.12122 | 2.64288 | 6.15952 | 2.69544 | 6.16305 |
| ADORA3 | 0 | 0.011493 | 0.095024 | 0.037209 | 0.109102 |
| P2RX1 | 0 | 0.054591 | 0.016488 | 0.027857 | 0 |
| P2RX2 | 0 | 0 | 0 | 0 | 0 |
| P2RX3 | 0 | 0 | 0 | 0 | 0.013077 |
| P2RX4 | 21.2695 | 17.1199 | 34.0617 | 17.4675 | 23.6657 |
| P2RX5 | 0 | 0.136975 | 2.810431 | 0.389678 | 0.881001 |
| P2RX6 | 4.67304 | 1.871812 | 0.760653 | 2.494822 | 0.731213 |
| P2RX7 | 3.773717 | 8.00451 | 1.279602 | 4.16289 | 0.460136 |
| P2RY1 | 0.575883 | 0.261818 | 3.38255 | 4.08394 | 2.41749 |
| P2RY2 | 0 | 0.010166 | 0.082483 | 0.01815 | 0.134645 |
| P2RY4 | 0 | 0.026812 | 0 | 0.023911 | 0.014776 |
| P2RY6 | 1.90573 | 0.029593 | 0.090574 | 0.162274 | 0.111065 |
| P2RY8 | 0 | 0.009783 | 0.039814 | 0 | 0.124417 |
| P2RY10 | 0 | 0 | 0 | 0 | 0 |
| P2RY11 | 0.916745 | 0 | 0.528882 | 0 | 0.610563 |
| P2RY12 | 0.352434 | 0.308527 | 0 | 0.011008 | 0 |
| P2RY13 | 0 | 0.007598 | 0 | 0 | 0 |
| P2RY14 | 0 | 0 | 0 | 0.00751 | 0 |
|  |  |  |  |  |  |
| ADRB2 | 2.10577 | 0.080434 | 5.75383 | 3.37604 | 8.5156 |

Supplementary Table 1 – Purinergic receptor RNA expression data from human airway smooth muscle (HASM) cells derived from 5 donors. Expression data are also provided for the beta-2 adrenergic receptor (ADRB2), a GPCR that is currently the target for the treatment of respiratory disease. RNA-Seq data are mean fragments per kilobase per million mapped reads (FPKM), where reads are normalized according to sequencing depth and gene length. Genes are considered expressed if FPKM > 1 across all donors.

|  | **HBEC (FPKM)** | | | | | | | |
| --- | --- | --- | --- | --- | --- | --- | --- | --- |
| **Receptor** | **Donor 1** | **Donor 2** | **Donor 3** | **Donor 4** | **Donor 5** | **Donor 6** | **Donor 7** | **Donor 8** |
| ADORA1 | 0.047768 | 0.035242 | 0.031915 | 0 | 0 | 0 | 0.021188 | 0 |
| ADORA2A | 0 | 0.010428 | 0.000798 | 0.014558 | 0 | 0.011747 |  |  |
| ADORA2B | 14.4149 | 16.5672 | 13.8563 | 13.0441 | 14.1632 | 14.337 | 18.4104 | 13.3512 |
| ADORA3 | 0 | 0 | 0 | 0 | 0 | 0 | 0 | 0 |
| P2RX1 | 0 | 0 | 0 | 0 | 0 | 0 | 0 | 0 |
| P2RX2 | 0 | 0 | 0 | 0 | 0 | 0 | 0 | 0.015425 |
| P2RX3 | 0 | 0 | 0 | 0 | 0 | 0 | 0 | 0 |
| P2RX4 | 5.07212 | 5.31907 | 6.72804 | 9.66426 | 4.62271 | 3.11025 | 4.52991 | 7.33128 |
| P2RX5 | 0.494951 | 0.493205 | 0.974534 | 0.652712 | 0.106072 | 0.621807 |  |  |
| P2RX6 | 0.224388 | 0.221469 | 0.278032 | 0.048462 | 0.559193 | 0.871078 |  |  |
| P2RX7 | 0.080824 | 0.18079 | 0.346432 | 0.127969 | 0.425487 | 0.104121 |  |  |
| P2RY1 | 3.57717 | 1.42244 | 3.81023 | 6.02538 | 5.15202 | 2.88723 | 2.15341 | 3.64287 |
| P2RY2 | 3.46546 | 3.129157 | 7.978928 | 3.635457 | 12.83917 | 12.00147 |  |  |
| P2RY4 | 0 | 0.035063 | 0.04886 | 0 | 0.083011 | 0.061404 | 0 | 0 |
| P2RY6 | 0.044897 | 0.085711 | 0.142668 | 0.020923 | 0.207678 | 0.41512 | 1.12576 | 0.501947 |
| P2RY8 | 0.006342 | 0 | 0 | 0 | 0 | 0 | 0 | 0 |
| P2RY10 | 0 | 0 | 0 | 0 | 0 | 0 | 0 | 0 |
| P2RY11 | 0 | 0.126242 | 0.617677 | 0 | 0.174722 | 0 |  |  |
| P2RY12 | 0 | 0 | 0 | 0 | 0 | 0 | 0 | 0 |
| P2RY13 | 0 | 0 | 0 | 0 | 0 | 0 | 0 | 0 |
| P2RY14 | 0.010788 | 0.015972 | 0 | 0 | 0 | 0 | 0 | 0 |
|  |  |  |  |  |  |  |  |  |
| ADRB2 | 8.73117 | 11.6577 | 12.217 | 9.35407 | 24.8322 | 32.7587 | 11.5319 | 14.1327 |

Supplementary Table 2 – Purinergic receptor RNA expression data from human bronchial epithelial cells (HBEC) derived from 8 donors. Expression data are also provided for the beta-2 adrenergic receptor (ADRB2), a GPCR that is currently the target for the treatment of respiratory disease. RNA-Seq data are mean fragments per kilobase per million mapped reads (FPKM), where reads are normalized according to sequencing depth and gene length. Genes are considered expressed if FPKM > 1 across all donors.

| **Donor number** | **Donor 1** | **Donor 2** | **Donor 3** | **Donor 4** | **Donor 5** |
| --- | --- | --- | --- | --- | --- |
| **Age** | 63 | 53 | 59 | 64 | 57 |
| **Sex** | Male | Male | Female | Male | Male |
| **Smoking status** | Ex-smoker | Ex-smoker | Ex-smoker | Heavy smoker | Ex-smoker |
| **Disease status/reason for operation** | COPD; Double lung transplant | COPD; Double lung transplant | Right upper lobe squamous cell carcinoma | Squamous cell carcinoma of right lung | Pulmonary fibrosis |
| **Tissue source** | Nottingham Biobank | Nottingham Biobank | Royal Papworth | Royal Papworth | Nottingham Biobank |

Supplementary Table 3 – Demographics for the human airway smooth muscle (HASM) cell donors used. HASM cells were obtained from lung tissue from these donors.

| **Donor number** | **Donor 1** | **Donor 2** | **Donor 3** | **Donor 4** | **Donor 5** | **Donor 6** | **Donor 7** | **Donor 8** |
| --- | --- | --- | --- | --- | --- | --- | --- | --- |
| **Age** | 56 | 50 | 19 | 43 | 19 | 18 | 60 | 38 |
| **Sex** | Male | Male | Male | Male | Female | Female | Female | Male |
| **Smoker** | Yes | Yes | No | No | No | No | No | No |

Supplementary Table 4 – Demographics for the human bronchial epithelial cell (HBEC) donors used. HBECs were purchased from Lonza. All donors were of Caucasian ethnicity.

**References**

HALL, R. J., O'LOUGHLIN, J., BILLINGTON, C. K., THAKKER, D., HALL, I. P. & SAYERS, I. 2021. Functional Genomics of GPR126 in airway smooth muscle and bronchial epithelial cells. *FASEB J.*
